# Impact of advanced Parkinson’s disease on caregivers: an international real-world study

**DOI:** 10.1007/s00415-022-11546-5

**Published:** 2023-01-12

**Authors:** Pablo Martinez-Martin, Matej Skorvanek, Tove Henriksen, Susanna Lindvall, Josefa Domingos, Ali Alobaidi, Prasanna L. Kandukuri, Vivek S. Chaudhari, Apeksha B. Patel, Juan Carlos Parra, James Pike, Angelo Antonini

**Affiliations:** 1 Center for Networked Biomedical Research, Neurodegenerative Diseases (CIBERNED), Carlos III Institute of Health, Madrid, Spain; 2 Department of Neurology, P. J. Šafárik University, Košice, Slovakia; 3 Department of Neurology, University Hospital L. Pasteur, Košice, Slovakia; 4 Movement Disorder Clinic, University Hospital of Bispebjerg, Copenhagen, Denmark; 5 European Parkinson’s Disease Association (EPDA), Sevenoaks, UK; 6 AbbVie Inc., North Chicago, IL USA; 7 University of Illinois at Chicago, Chicago, IL USA; 8 Adelphi Real World, Adelphi Mill, Bollington, UK; 9 Movement Disorders Unit, Department of Neuroscience, University of Padova, Padua, Italy

**Keywords:** Advanced Parkinson’s disease, Caregiver burden, Zarit Burden interview, EuroQol 5 dimension, Quality of life

## Abstract

**Background:**

Caring for a partner or family member with Parkinson’s disease (PD) negatively affects the caregiver’s own physical and emotional well-being, especially those caring for people with advanced PD (APD). This study was designed to examine the impact of APD on caregiver perceived burden, quality of life (QoL), and health status.

**Methods:**

Dyads of people with PD and their primary caregivers were identified from the Adelphi Parkinson’s Disease Specific Program (DSP™) using real-world data from the United States, Japan and five European countries. Questionnaires were used to capture measures of clinical burden (people with PD) and caregiver burden (caregivers).

**Results:**

Data from 721 patient-caregiver dyads in seven countries were captured. Caregivers had a mean age 62.6 years, 71.6% were female, and 70.4% were a spouse. Caregivers for people with APD had a greater perceived burden, were more likely to take medication and had lower caregiver treatment satisfaction than those caring for people with early or intermediate PD; similar findings were observed for caregivers of people with intermediate versus early PD. Caregivers for people with intermediate PD were also less likely to be employed than those with early PD (25.3% vs 42.4%) and spent more time caring (6.6 vs 3.2 h/day).

**Conclusions:**

This real-world study demonstrates that caregivers of people with APD experience a greater burden than those caring for people with early PD. This highlights the importance of including caregiver-centric measures in future studies, and emphasizes the need for implementing treatments that reduce caregiver burden in APD.

**Trial registration:** N/A.

## Background

Parkinson’s disease (PD) is a progressive neurodegenerative disorder, characterized by tremor, bradykinesia and rigidity, with postural instability commonly seen later in the disease [1]. Considerable evidence demonstrates that PD has a profound impact on the quality of life (QoL) of patients, as well as their ability to carry out activities of daily living (ADLs) [2–12]. The incidence of PD has been estimated to range from five new cases in 100,000 to over 35 in 100,000 per year [13, 14], with both incidence and prevalence of PD increasing with age [15]. The number of cases of PD is predicted to increase from 6 million in 2015 to more than 12 million by 2040 [16].

The increasing prevalence of PD will have substantial implications for healthcare systems and economies worldwide, as people with PD tend to have high medical care needs, reduced productivity, and a dependence on informal and professional caregivers [17]. For example, an analysis based on multiple sources in the United States (US) estimated a total economic PD burden of $51.9 billion in 2017, projected to surpass $79 billion by 2037 [17].

Most people with PD live in the community and are looked after by informal caregivers such as spouses, adult children, friends, or other nonpaid individuals [18, 19]. However, caring for a partner or family member with PD negatively affects the caregiver’s own physical and emotional psychosocial well-being, and worsens QoL [2, 20–30]. Furthermore, the burden on caregivers tends to increase with advancing disease, along with deterioration in the speech and cognitive abilities of the person with PD [31–33].

In addition to the mental impact of caring for someone with PD, caregivers also often experience an economic burden, and sometimes financial hardship [34]. Added to the increased financial cost of caring for someone with PD, a caregiver is also likely to experience reduced productivity at work through absenteeism, disability, or forced retirement. Estimates show that PD caregivers have a higher cumulative income loss over 5 years compared with control subjects ($5967 vs $2634 by year 5; *p* = 0.03) [34].

The increasing prevalence of PD predicted over the next 20 years will compound each of these issues, placing further burden on healthcare systems, and increasing the financial, mental, and physical strain on caregivers. The problem for caregivers is more acute as pressures on central healthcare resources will increase the reliance on informal care at home [35]. Despite this impending crisis, little research has been undertaken to quantify the increased burden experienced by caregivers with advanced PD (APD), and the impact this has on the caregiver’s ability to care, own well-being, and QoL. The purpose of this study was to examine the impact of APD on caregiver perceived burden, QoL, and health status, using real-world data from the US, Japan and five European countries.

## Methods

Data for this study were drawn from the Adelphi Parkinson’s Disease Specific Program (DSP™), a point-in-time survey of physicians and their consulting PD patients presenting in a real-world clinical setting between 2017 and 2019. Dyads of people with PD and their primary caregivers were identified from the Adelphi DSP in five European countries (France, Germany, Italy, Spain, UK), the US, and Japan. The DSP methodology has been previously published and validated [36]. In brief, participating physicians completed a record form for the next 12 consecutively consulting eligible patients, and each person with PD was then invited to complete a patient-reported questionnaire. Caregivers were also asked to complete a form, reporting on their own demographic characteristics and their perceived burden of care. Validated translations of the survey materials were developed for each of the countries where data collection took place.

Respondents provided informed consent for use of their data; all data were aggregated and de-identified before receipt. Surveys were conducted in full accordance with the US Health Insurance Portability and Accountability Act of 1996. The study protocol was approved by the Western Institutional Review Board (Puyallup, Washington, US) on 3 July 2019. All data were collected according to the requirements of ethics committee approval, including obtaining participants’ informed consent.

### Participants

Physicians were eligible to participate in the survey if they were a neurologist experienced in the management of people with movement disorders, and were personally responsible for the treatment and management of three or more people with PD per week. Individuals were eligible for inclusion if they were diagnosed with PD on or before the date of their last consultation (i.e., at the time of data collection), were aged ≥ 18 years, were not currently involved in any clinical trials, and had a caregiver who was also willing to participate. Participants were classified by their physician as having early, intermediate, or advanced Parkinson’s disease (APD) based on their best clinical judgment after considering overall patient history including demographics, clinical characteristic, concomitant medications, and other patient-reported outcomes.

### Measures

Demographic and disease characteristics of enrolled participants were captured. Measures of clinical burden included the physician’s best clinical judgment of disease severity (early PD, intermediate PD or APD), Charlson Comorbidity Index score (CCI; based on a number of conditions that are each assigned an integer weight from one to six; higher score indicates greater comorbidity burden) [37], time since diagnosis, Hoehn and Yahr scale (HY; measure of progression of symptoms and disability in PD; higher score indicates greater burden) [38], off-time, dyskinesia, axial symptoms (uncontrolled shuffling walk, freezing of gait, and falling/imbalance), Unified Parkinson’s Disease Rating Scale (UPDRS; clinical rating scale for PD encompassing behavior and mood, ADLs, motor symptoms, and complications; higher score indicates greater burden) [39], and Mini-Mental State Examination (MMSE; 11-question instrument testing five areas of cognitive function; lower score indicates worse cognitive function) [40].

Caregiver characteristics captured included demographic details, relationship to participant, duration of caring responsibilities, hours per day spent caring, caregiver medication use to support PD-related caregiving, 3-level version of the EuroQol 5 Dimension (EQ-5D-3L; a generic instrument to assess health-related QoL; scores range from 0 to 1, with a lower score indicating poorer QoL) [41], Zarit Burden Interview (ZBI; caregiver self-report 29-item questionnaire; higher score indicates greater burden) [42]. Categories of ZBI were interpreted as follows: slight burden (ZBI score 0–20), mild burden (ZBI score 21–40), moderate burden (ZBI score 41–60), and severe burden (ZBI score 61+) [43], and satisfaction with PD treatment (linear scale from 1 [very unsatisfied] to 7 [very satisfied]).

### Statistical analysis

Demographic data of people with PD and caregivers were analyzed descriptively. Participant clinical characteristics, and measures of caregiver burden were also analyzed descriptively. Group comparisons were conducted using analysis of variance (ANOVA), chi-squared, and Kruskal–Wallis tests. Incremental caregiver burden was evaluated using generalized linear models (Gaussian, with identity link), the partial proportional odds model, and logistic regression models (reference = early PD) adjusting for country, participant factors (age, sex, CCI), and caregiver factors (age, sex, marital status). Pairwise comparisons were made using Sidak’s method to adjust for multiple comparisons. All analyses were conducted in Stata v17.0 (StataCorp LLC, College Station, Texas, USA).

## Results

### Patient demographics

In total, 222 physicians enlisted 721 patient-caregiver dyads from seven countries, with Germany accounting for the highest percentage of people with PD (23.2%). The mean (SD) age of people with PD at enrollment was 70.7 (9.6) years, and the mean age (SD) at diagnosis was 65.2 (9.7 years). Most (61.9%) people with PD were male, and the mean (SD) CCI score was 0.5 (1.1).

One hundred and twenty-seven participants (17.6%) were classified as having APD, with 258 (35.8%) and 336 (46.6%) recorded as having early and intermediate disease, respectively. The demographic and disease characteristics of enrolled participants are summarized in Table 1.

**Table 1:** Clinical characteristics of enrolled participants

	Overall (*n* = 721)^a^	Early (*n* = 258)^a^	Intermediate (*n* = 336)^a^	Advanced (*n* = 127)^a^	*p*-value
Country, *n* (%)					< 0.0001
France	88 (12.2)	46 (17.8)	38 (11.3)	4 (3.1)	
Germany	167 (23.2)	73 (28.3)	72 (21.4)	22 (17.3)	
Italy	25 (3.5)	9 (3.5)	9 (2.7)	7 (5.5)	
Spain	106 (14.7)	48 (18.6)	45 (13.4)	13 (10.2)	
UK	76 (10.5)	29 (11.2)	38 (11.3)	9 (7.1)	
US	152 (21.1)	19 (7.4)	76 (22.6)	57 (44.9)	
Japan	107 (14.8)	34 (13.2)	58 (17.3)	15 (11.8)	
Age					< 0.0001
Mean (SD)	70.7 (9.6)	66.7 (10.0)	72.1 (8.4)	75.0 (8.6)	
≥ 65 years, *n* (%)	567 (78.6)	156 (60.5)	297 (88.4)	114 (89.8)	
Age at diagnosis					0.0893
*n*	583	213	278	92	
Mean (SD)	65.2 (9.7)	64.1 (10.1)	65.7 (9.1)	66.3 (10.2)	
Sex, *n* (%)					0.0620
Male	446 (61.9)	145 (56.2)	220 (65.5)	81 (63.8)	
Ethnicity, *n* (%)					0.2516
African American	12 (1.7)	2 (0.8)	4 (1.2)	6 (4.7)	
Afro-Caribbean	1 (0.1)	1 (0.4)	0 (0.0)	0 (0.0)	
Asian–Indian subcontinent	8 (1.1)	3 (1.2)	4 (1.2)	1 (0.8)	
Asian–other	2 (0.3)	1 (0.4)	1 (0.3)	0 (0.0)	
Chinese	1 (0.1)	0 (0.0)	1 (0.3)	0 (0.0)	
Hispanic/Latina/Latino	4 (0.6)	2 (0.8)	2 (0.6)	0 (0.0)	
Japanese	106 (14.7)	33 (12.8)	58 (17.3)	15 (11.8)	
Middle Eastern	5 (0.7)	3 (1.2)	2 (0.6)	0 (0.0)	
Mixed race	4 (0.6)	2 (0.8)	0 (0.0)	2 (1.6)	
White/Caucasian	577 (80.0)	211 (81.8)	263 (78.3)	103 (81.1)	
Other	1 (0.1)	0 (0.0)	1 (0.3)	0 (0.0)	
Charlson comorbidity index					< 0.0001
Mean (SD)	0.5 (1.1)	0.3 (0.8)	0.5 (1.0)	1.0 (1.5)	
Time since diagnosis (years)^a^					< 0.0001
*n*	583	213	278	92	
Mean (SD)	5.1 (4.9)	2.0 (2.3)	6.1 (4.6)	9.1 (5.8)	
Hoehn and Yahr, *n* (%)					< 0.0001
*n*	721	258	336	127	
1	172 (23.9)	144 (55.8)	26 (7.7)	2 (1.6)	
2	232 (32.2)	94 (36.4)	131 (39.0)	7 (5.5)	
3	179 (24.8)	17 (6.6)	127 (37.8)	35 (27.6)	
4	125 (17.3)	2 (0.8)	49 (14.6)	74 (58.3)	
5	13 (1.8)	1 (0.4)	3 (0.9)	9 (7.1)	
Off-time, h/day					< 0.0001
*n*	678	250	309	119	
Mean (SD)	1.0 (1.7)	0.1 (0.4)	1.1 (1.9)	2.4 (1.6)	
Dyskinesia, h/day					< 0.0001
*n*	685	256	310	119	
Mean (SD)	0.3 (0.9)	0.0 (0.3)	0.3 (1.0)	0.9 (1.3)	
Axial symptoms, *n* (%)					
Shuffling walk	374 (51.9)	78 (30.2)	203 (60.4)	93 (73.2)	< 0.0001
Freezing of gait	257 (35.6)	36 (14.0)	134 (39.9)	87 (68.5)	< 0.0001
Falling/imbalance	273 (37.9)	46 (17.8)	148 (44.0)	79 (62.2)	< 0.0001
UPDRS score					< 0.0001
*n*	144	63	66	15	
Mean (SD)	38.7 (29.0)	25.0 (16.9)	43.2 (28.3)	76.1 (34.1)	
MMSE score					< 0.0001
*n*	170	40	92	38	
Mean (SD)	25.0 (4.0)	27.8 (2.5)	24.9 (3.5)	22.3 (4.3)	

*MMSE* mini-mental state examination; *SD* standard deviation; *UPDRS* unified Parkinson’s disease rating scale

^a^Number of participants, unless otherwise stated

### Caregiver demographics

The majority (70.4%) of caregivers enrolled in this study were the spouse of the person with PD, and most (71.6%) were female. The mean (SD) age of caregivers was 62.6 (12.8) years, and they had been caring for the person with PD for a mean (SD) of 4.6 (4.2) years. The characteristics of the caregivers are summarized in Table 2.

**Table 2:** Characteristics of enrolled caregivers

	Overall (*n* = 721)^a^	Early (*n* = 258)^a^	Intermediate (*n* = 336)^a^	Advanced (*n* = 127)^a^	*p*-Value
Relationship to participant, *n* (%)					0.0042
*n*	720	257	336	127	
Partner/spouse	507 (70.4)	192 (74.7)	243 (72.3)	72 (56.7)	
Child	169 (23.5)	49 (19.1)	75 (22.3)	45 (35.4)	
Other	44 (6.1)	16 (6.2)	18 (5.4)	10 (7.9)	
Sex, *n* (%)					0.0197
Male	205 (28.4)	86 (33.3)	94 (28.0)	25 (19.7)	
Marital status, *n* (%)					0.1152
n	619	242	278	99	
Married/widowed	556 (89.8)	216 (89.3)	256 (92.1)	84 (84.8)	
Single/separated/divorced/other	63 (10.2)	26 (10.7)	22 (7.9)	15 (15.2)	
Employment status, *n* (%)					< 0.0001
*n*	708	255	328	125	
Not currently employed	478 (67.5)	147 (57.6)	245 (74.7)	86 (68.8)	
Currently employed	230 (32.5)	108 (42.4)	83 (25.3)	39 (31.2)	
Age					0.0417
*n*	718	257	335	126	
Mean (SD)	62.6 (12.8)	61.0 (12.8)	63.7 (12.7)	62.8 (12.9)	
Country, *n* (%)					< 0.0001
France	88 (12.2)	46 (17.8)	38 (11.3)	4 (3.1)	
Germany	167 (23.2)	73 (28.3)	72 (21.4)	22 (17.3)	
Italy	25 (3.5)	9 (3.5)	9 (2.7)	7 (5.5)	
Spain	106 (14.7)	48 (18.6)	45 (13.4)	13 (10.2)	
UK	76 (10.5)	29 (11.2)	38 (11.3)	9 (7.1)	
US	152 (21.1)	19 (7.4)	76 (22.6)	57 (44.9)	
Japan	107 (14.8)	34 (13.2)	58 (17.3)	15 (11.8)	
Length of time been caring for person for PD (years)					< 0.0001
*n*	576	205	266	105	
Mean (SD)	4.6 (4.2)	2.8 (2.6)	5.5 (4.6)	5.9 (4.7)	
Hours per day caring for person with PD (assumed 16 maximum)					< 0.0001
*n*	390	136	192	62	
Mean (SD)	5.7 (6.1)	3.2 (4.7)	6.6 (6.2)	8.4 (6.5)	

*PD* Parkinson’s disease; *SD* standard deviation

^a^Caregiver number unless otherwise stated

### Impact of disease severity on caregiver productivity

**Fig. 1:**
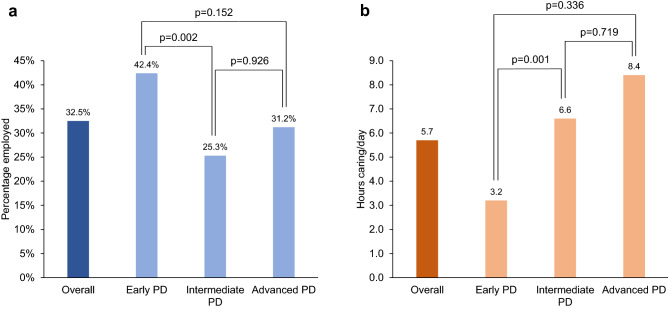
**a** Percentage of caregivers currently employed by PD severity. **b** Number of hours per day caring for person with PD by PD severity. *p*-values for pairwise comparisons using Sidak’s method to adjust for multiple comparisons. *PD* Parkinson’s disease

A lower percentage of caregivers for people with intermediate PD were currently employed compared with caregivers for people with early PD (25.3% vs 42.4%; *p* < 0.001). However, the proportion of employed caregivers was numerically higher for people with APD than intermediate PD (31.2% vs 25.3%; *p* = NS) (Fig. 1a). This mirrors the age profile for caregivers, whereby the care-givers for people with early PD were younger than those for intermediate PD, while caregivers for people with APD were also younger than those for intermediate PD (Table 2).

The number of hours per day spent caring for the person with PD was significantly greater in caregivers of people with intermediate PD (6.6 [6.2] h/day) compared with those caring for individuals with early PD (3.2 [4.7] h/day; *p* = 0.001) (Fig. 1b). Caregivers of people with APD spent 8.4 (6.5) h caring per day (*p* = 0.336 vs early PD and *p* = 0.719 vs intermediate PD).

### Impact of disease severity on caregiver burden

After adjusting for confounders, caregivers of people with intermediate PD had significantly higher self-perceived burden compared with those caring for people with early PD (ZBI = 29.5 vs 24.7; *p* < 0.001). In addition, caregivers of people with APD had significantly higher self-perceived burden (ZBI = 34.5) than those caring for people with intermediate PD (*p* = 0.005) or early PD (*p* < 0.001) (Table 3).

**Table 3:** Caregiver burden by Parkinson’s disease severity

Measure	Early PD (*n* = 258)	Intermediate PD (*n* = 336)	Advanced PD (*n* = 127)	Pairwise comparisons^a^
Caregiver perceived burden (ZBI Score)	24.7 ± 17.1	29.5 ± 17.0	34.5 ± 17.6	Early vs intermediate: *p* < 0.001 Intermediate vs advanced: *p* = 0.005 Early vs advanced: *p* < 0.001
Mean difference (95% CI)^b^	Reference	6.5 (2.8, 10.2)	13.0 (7.6, 18.3)	
Medication intake due to caregiving (Yes)	13.8%	25.2%	34.7%	Early vs intermediate: *p* = 0.037 Intermediate vs advanced: *p* = 0.017 Early vs advanced: *p* < 0.001
Absolute % difference (95% CI)^c^	Reference	9.1% (0.0, 17.8)	25.2% (10.7, 39.8)	
Caregiver quality of life EQ-5D-3L score	0.91 ± 0.14	0.88 ± 0.17	0.87 ± 0.20	Early vs intermediate: *p* = 0.190 Intermediate vs advanced: *p* = 0.789 Early vs advanced: *p* = 0.121
Mean difference (95% CI)^b^	Reference	− 0.03 (− 0.06, 0.01)	− 0.04 (− 0.10, 0.01)	
Caregiver treatment satisfaction^d^	5.3 ± 1.3	4.9 ± 1.1	4.4 ± 1.4	Early vs intermediate: *p* = 0.003 Intermediate vs advanced: *p* < 0.001 Early vs advanced: *p* < 0.001
Mean difference (95%)^b^	Reference	− 0.39 (− 0.67, − 0.10)	− 1.03 (− 1.45, − 0.61)	

*CI* Confidence Interval; *OR* Odds ratio; *ZBI* Zarit Burden Interview

^a^Pairwise comparisons calculated using Sidak’s method to adjust for multiple comparisons

^b^Generalized linear model (Gaussian, with identity link) adjusted for country, patient factors (age, sex, Charlson comorbidity index), and caregiver factors (age, sex, marital status)

^c^Logistic regression model adjusted for country, patient factors (age, sex, Charlson comorbidity index), and caregiver factors (age, sex, marital status)

^d^Linear scale (1 = very unsatisfied; 7 = very satisfied)

Similarly, caregivers of people with intermediate PD were significantly more likely to take medication due to PD-related caregiving than those caring for people with early PD (25.2% vs 13.8%, respectively, of caregivers took medication; *p* = 0.037). More than one-third (34.7%) of caregivers of people with APD took medication, which was significantly more than among caregivers of people with intermediate PD (*p* = 0.017) or early PD (*p* < 0.001) (Table 3).

A similar pattern was found for caregiver treatment satisfaction, which was significantly lower among caregivers of people with intermediate PD compared with those caring for an individual with early PD (4.9 vs 5.3 on a linear scale where 1 = very unsatisfied and 7 = very satisfied; *p* = 0.003), and for APD compared with intermediate PD (4.4 vs 4.9; *p* < 0.001). Caregivers of people with APD also had a significantly lower caregiver treatment satisfaction than those of people with early PD (*p* < 0.001) (Table 3).

In terms of caregiver QoL as assessed by the EQ-5D-3L score, no pairwise significant differences were found between caregivers of people with APD, intermediate PD, or early PD (Table 3).

**Fig. 2:**
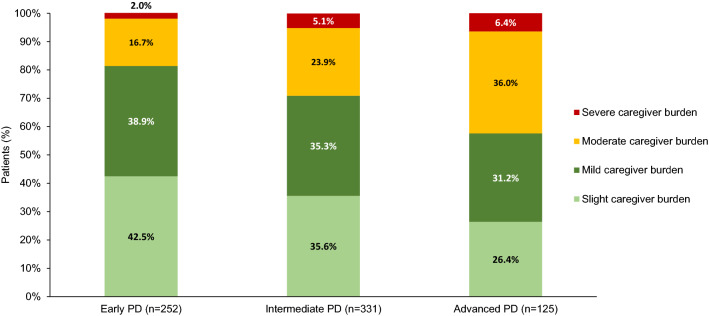
Caregiver perceived burden by Parkinson’s disease severity

As assessed by the ZBI, the proportion of caregivers experiencing moderate burden increased significantly with severity of PD (16.7%, 23.9%, and 36.0%, respectively; Sidak adjusted *p* = 0.001 for early vs intermediate PD; *p* = 0.007 for intermediate vs APD; and *p* < 0.001 for early vs APD). A similar increase with PD severity was observed among caregivers experiencing severe burden (2.0%, 5.1%, 6.4%, respectively; *p* = 0.006 for early vs intermediate PD; *p* = 0.045 for intermediate vs APD; and *p* = 0.002 for early vs APD) (Fig. 2).

## Discussion

As pressure on healthcare resources increases, more individuals with chronic diseases are being cared for in the home, with the burden of care falling on family members (particularly a spouse) [35, 44, 45]. As PD is largely a disease of older age, it is likely that a person with PD will be cared for by a spouse who is him or herself elderly, and may have other comorbid conditions [46, 47]. Thus, national healthcare systems are being protected from a substantial amount of time and resource that is currently being provided by informal caregivers [48].

This study uses real-world data from 721 patient–caregiver dyads in seven countries to quantify the burden on caregivers, and particularly to evaluate the burden that falls on caregivers of people with APD. With a mean age 62.6 years, and a greater likelihood of being female (71.6%) and a spouse (70.4%), the profile of caregivers in this study is consistent with that reported in previous studies [35, 44–47].

One key finding of this study is that someone looking after a person with APD had significantly higher perceived burden, was significantly more likely to start medications due to PD-related caregiving, and was significantly less likely to be satisfied with treatment compared with the caregiver of someone with early or intermediate PD. Similar findings were also found for caregivers of people with intermediate PD compared with those looking after someone with early PD. Interestingly, no significant pairwise differences were observed for caregiver generic QoL according to severity of PD, as measured by the EQ-5D-3L score.

The range of ZBI scores observed in this study (24.7–34.5, depending on PD severity) are largely consistent with previous reports, which have also reported a greater burden with more severe PD [24, 46, 49–51]. The mean EQ-5D-3L score of 0.9 reported here irrespective of severity also suggests a slightly greater effect on caregiver QoL in the current study compared with previously reported studies reporting an EQ-5D-3L score of 0.7 [51, 52].

The amount of time required for caring also increased between intermediate and early PD (3.2 vs 6.6 h/day; *p* = 0.001); consistent with previous studies [47, 53]. However, while caregivers of people with intermediate PD were significantly less likely to be employed than caregivers of people with early PD, a numerically greater (but statistically non-significant) proportion of caregivers of people with advanced than intermediate PD were employed. This is initially surprising, as people with APD tended to be older than those with intermediate or early PD, suggesting that their spouses may also be older. However, people with more advanced disease are more likely to rely on professional care, including residential care, potentially allowing more time for a caregiver to work outside the home. In addition, there was a greater percentage of children caring for people with APD (35.4%) compared with intermediate (22.3%) and early PD (19.1%), which may reflect the greater age of the patients themselves.

The burden on caregivers also depends on the specific characteristics of people with PD, with improved spiritual well-being [46], HRQoL [46], and mood [54] reducing caregiver burden, and prolonged disease duration [53] increasing the burden. Increasing motor symptoms [53, 54], overall disability, and greater HY score have also been shown to be correlated with caregiver burden [47]. Interestingly, non-motor symptoms appear to have a greater effect on caregiver burden than motor symptoms [47, 54–57], as they disproportionately increase the need for supervision, and affect the emotional relationship between person with PD and the caregiver [47].

A failure to intervene to support families caring for an individual with APD is likely to result in a greater healthcare burden, as the caregivers themselves become ill, unable to offer support, and require care of their own. The development of strategies to reduce the burden of caregivers of people with PD are thus urgently required. Suggestions under consideration include more home-based community-centered integrated care [58], home adaptation [59], financial support for caregivers [59], and targeted interventions and counseling to help caregivers manage stress [59, 60]. In addition, health and wellness checks for caregivers may be effective in mitigating risk of future decline in caregiver QoL [60]. Offering caregiver interventions relatively early in the course of PD may mitigate decline in QoL as well as alter the trajectory of caregiver functioning [60]. Furthermore, experience during the coronavirus disease 2019 (COVID-19) pandemic has demonstrated that the use of remote consultations and the availability of digital communications can also offer access to care in difficult circumstances [61–63]. Use of this technology may allow caregivers to receive ongoing support and medical intervention, even if they are not easily able to leave the house.

From the side of the person with PD, new treatments are urgently required to optimize symptom control, allowing people with PD to function to a higher level without the need for substantial caregiver support [64]. Development of new treatments for PD has been slow over recent years, and available therapies have focused on slowing symptomatic decline rather than addressing the underlying pathophysiology of PD [65]. However, interest in disease-modifying therapies (DMTs) is currently flourishing [65], with exciting therapeutic approaches including cellular therapies, immunotherapies, and vaccines, as well as repurposed drugs [66]. Given the number of different approaches currently in the treatment pipeline, there may be grounds for cautious optimism that more effective treatments will start to become available in the near future. Indeed, evidence already suggests that the availability of advanced therapies allows caregivers more time for themselves, improves QoL, and tends to improve mood compared with standard PD therapies [24, 67]. In particular, an observational prospective study demonstrated that 6 months’ use of levodopa/carbidopa intestinal gel among people with APD significantly improved the QoL and SF-36 health status of caregivers [68]. Another study demonstrated that 12 months of use significantly improved MCSI outcomes among caregivers [69]. A systematic review of studies investigating the effects of deep brain stimulation revealed some variation in studies regarding the impact of this intervention on caregiver burden [70].

This large, international dataset is derived from the Parkinson’s DSP, which is validated for capturing large, statistically robust samples of global real-world evidence. The strengths of this study lie in its basis in real-world clinical practice, while the pairing of caregivers and people with PD allows us to investigate the impact of patient characteristics on the caregiver burden. In particular, this study demonstrates that in the real world, caregivers of people with APD are more likely to be female, spend more hours caring per day, have a higher self-perceived burden, worse QoL and a greater reliance on medication compared with those caring for people with early PD. Although the lack of restrictions and control provide us with true real-world insights, they may also be considered limitations of the study. For example, the risk of bias and confounding factors due to the absence of randomization, risk of missing or misclassified data, and the self-reported nature of the caregiver data are potential deficiencies of the study. As the DSP methodology required caregivers to be present at physician consultation, there is a risk of bias towards those most actively involved in caring for the patient. Situations where caregivers avoid caring for the individual with PD, or other complex interactions of this nature, may be unaccounted for. In addition, there was an imbalance in the geographic distribution of participants included in this study, while the caring setting was not captured in the US and Japan. There is also the risk of recall bias when caregivers were asked about their recent experience of caring for the person with PD. One further limitation of the study was the use of physician’s opinion of disease severity. However, as this judgement was based on the overall condition of the patient, this was considered the best reflection of management in clinical practice. Based on the inclusion criteria for physicians it was felt that all were highly experienced and could reasonably be expected to reliably distinguish degree of PD severity.

In the future, additional data should be collected on the value of educational programs for caregivers of people with PD, in helping them understand the nature of the disease and its progressive nature. In addition, it would be useful to evaluate the potential role of online technology in supporting the caregivers of people with APD, to assess whether this approach can provide meaningful access to healthcare for caregivers unable to travel or leave their family member unattended. Finally, future studies should evaluate the impact of optimal PD symptom control on alleviating the burden of caregiving.

## Conclusions

This real-world study demonstrated that caregivers of people with advancing PD are more likely to be female, spend more hours caring per day, have a higher self-perceived burden, and a greater reliance on medication compared with those caring for people with early PD. These findings emphasize the importance of including caregiver-centric measures in future clinical study designs, and highlight the urgent need for new treatment options that lower burden among caregivers of people with advanced PD.

## Data Availability

The datasets used and/or analyzed during the current study are available from the corresponding author on reasonable request.

## Abbreviations

CCI: Charlson comorbidity index
DMT: Disease-modifying therapies
DSP: Disease specific program
HY: Hoehn and Yahr
MDS: Movement disorder society
MIUR: Ministry of Education University and Research
PD: Parkinson’s disease
QoL: Quality of life
UPDRS: Unified Parkinson’s disease rating scale
US: United States
ZBI: Zarit Burden interview
